# Supplementary material for: Investigating the Viability of Epithelial Cells on Polymer Based Thin-Films
Source: Polymers (Basel). 2021 Jul 14;13(14):2311. doi: 10.3390/polym13142311 (PMC8309445; doi:10.3390/polym13142311)
Supplement: Supplementary file 1 [file polymers-13-02311-s001.zip › polymers-1300035-supplementary.pdf]

polymers

MDPI

Article

Investigating the Viability of Epithelial Cells on Polymer Based Thin-Films

Boštjan Vihar [1,2,*], Jan Rožanc [1], Boštjan Krajnc [1], Lidija Gradišnik [1], Marko Milojević [1], Laura Činč Ćurić [1] and Uroš Maver [1,3]

[1] Faculty of Medicine. Institute of Biomedical Sciences. University of Maribor. Taborska ulica 8. SI-2000 Maribor. Slovenia; jan.rozanc@um.si (J.R.); bostjan.krajnc@um.si (B.K.); lidija.gradisnik@um.si (L.G.); marko.milojevic1@um.si (M.M.); laura.cinc@um.si (L.Č.Ć.); uros.maver@um.si (U.M.)
[2] IRNAS d.o.o. Valvasorjeva 42. 2000 Maribor. Slovenia
[3] Department of Pharmacology. Faculty of Medicine. University of Maribor. Taborska ulica 8. SI-2000 Maribor. Slovenia
* Correspondence: bostjan.vihar@um.si

Here additional results are presented, which were gathered for the preparation of the main manuscript.

Citation: Vihar. B.; Rožanc. J.; Krajnc. B.; Gradišnik. L.; Milojević. M.; Činč Ćurić. L.; Maver, M. Investigating the Viability of Epithelial Cells on Polymer Based Thin-Films. *Polymers* **2021**. *13*. 2311. https://doi.org/10.3390/polym13142311

Academic Editor(s): Ana Vallés-Lluch; Guillermo Vilariño-Feltrer; José Carlos Rodríguez Hernández

Received: date: 30 June 2021
Accepted: 9 July 2021
Published: 14 July 2021

Publisher's Note: MDPI stays neutral with regard to jurisdictional claims in published maps and institutional affiliations.

Copyright: © 2021 by the authors. Licensee MDPI. Basel. Switzerland. This article is an open access article distributed under the terms and conditions of the Creative Commons Attribution (CC BY) license (http://creativecommons.org/licenses/by/4.0/).

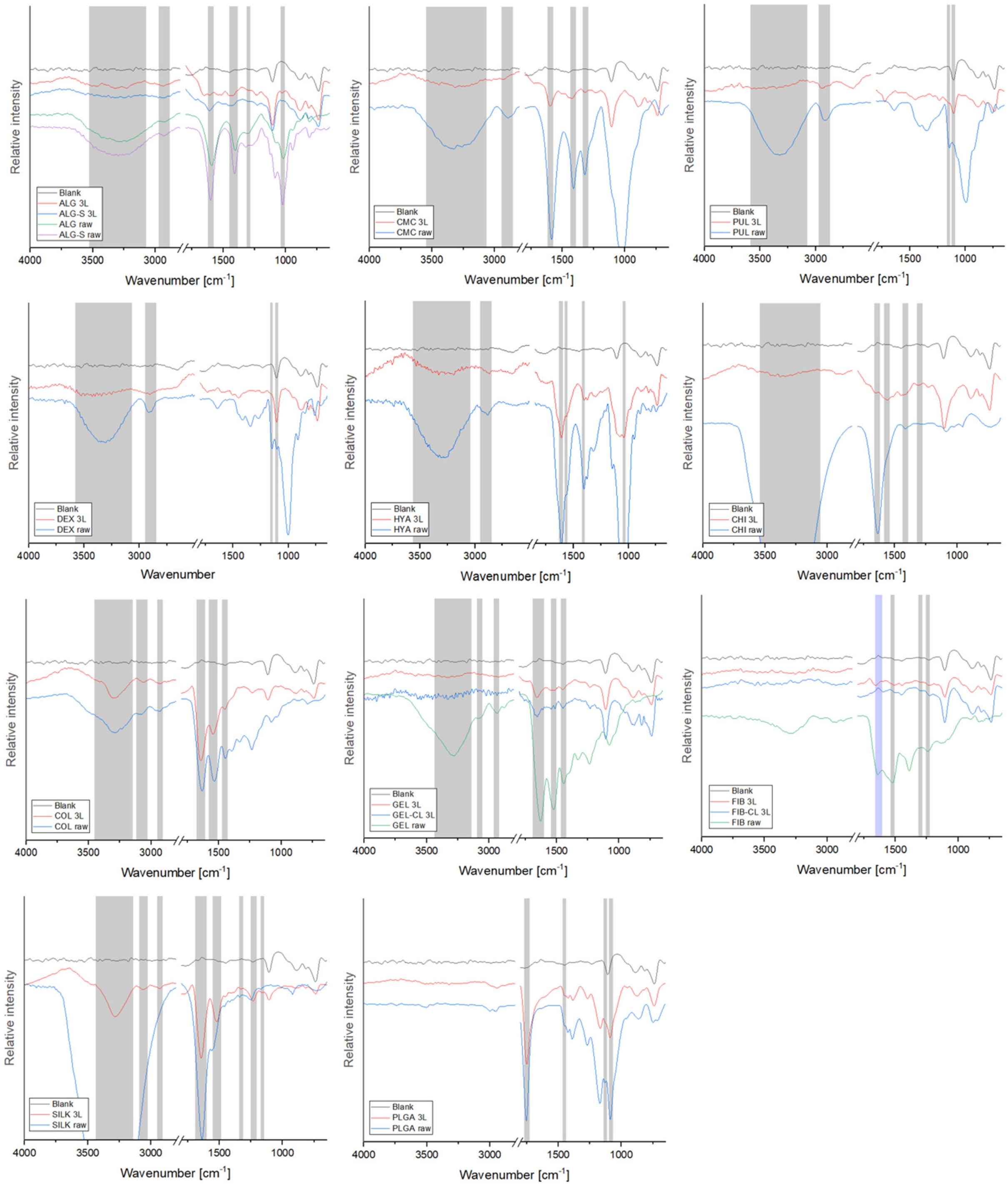

Figure S1. FTIR spectra of prepared thin films.

Figure S1 shows all FTIR recordings of thin films, compared to blank Si wafers and raw polymers.

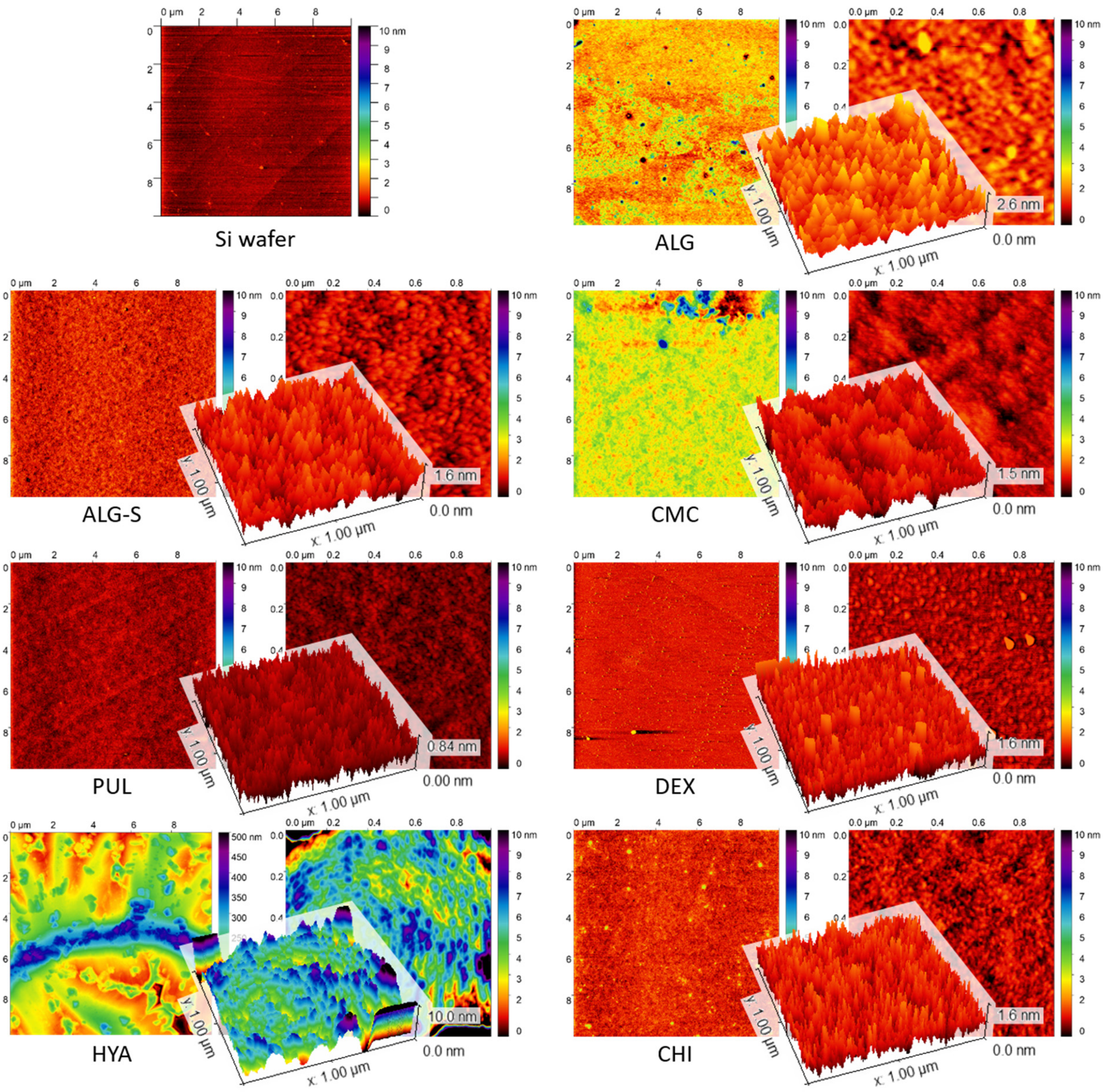

Figure S2.1. AFM scans of carbohydrate thin films.

Figure S2.1 shows all AFM recordings of carbohydrate thin films, compared to blank silicon wafers. For each sample a 10x10 μm scan, a 1x1 μm scan and a 3D rendering are shown.

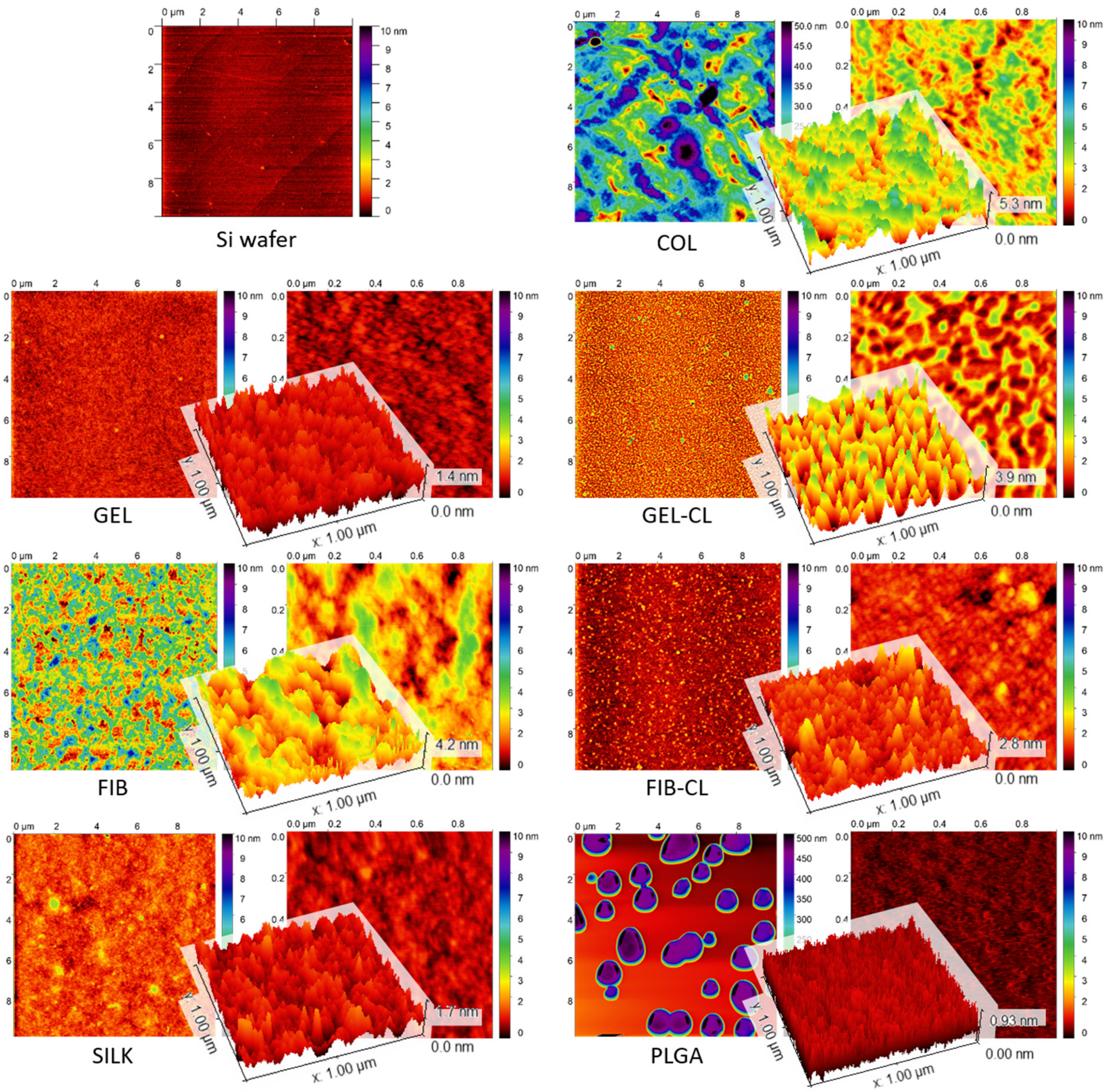

Figure S2.2 AFM scans of protein and PLGA thin films.

Figure S2.2 shows all AFM recordings of protein and PLGA thin films, compared to blank silicon wafers. For each sample a 10x10 µm scan, a 1x1 µm scan and a 3D rendering are shown.

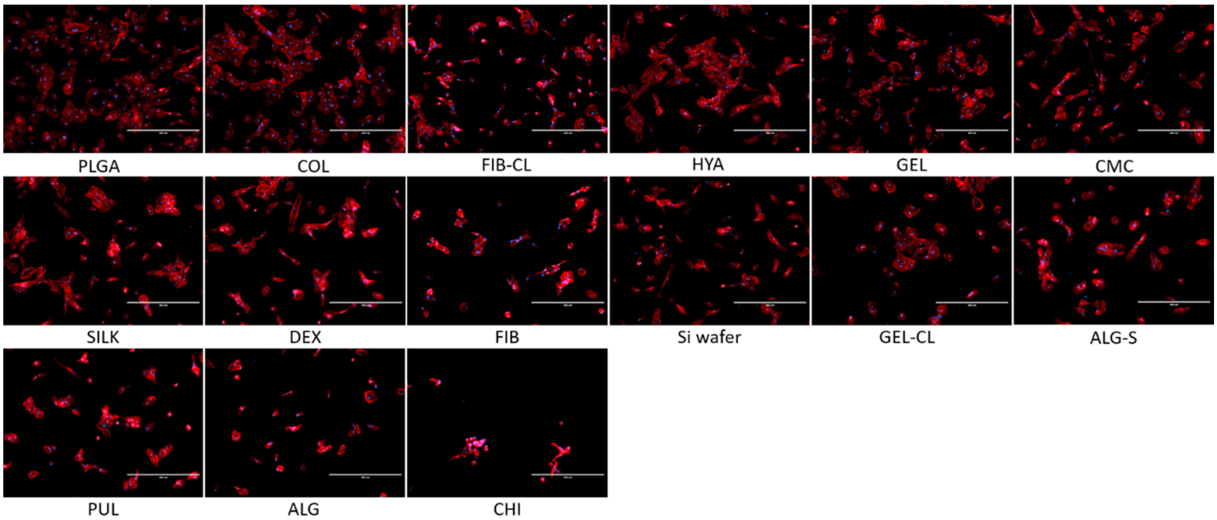

Figure S3. HUVEC cell culture images – ranked.

Figure S3 shows fluorescence micrographs (stained with DAPI and Phalloidin) of HUVEC cells cultured on polymer thin films, ordered according to biocompatibility score.

HUIEC cell culture images (stained with DAPI and Phalloidin) – ranked according to biocompatibility score.

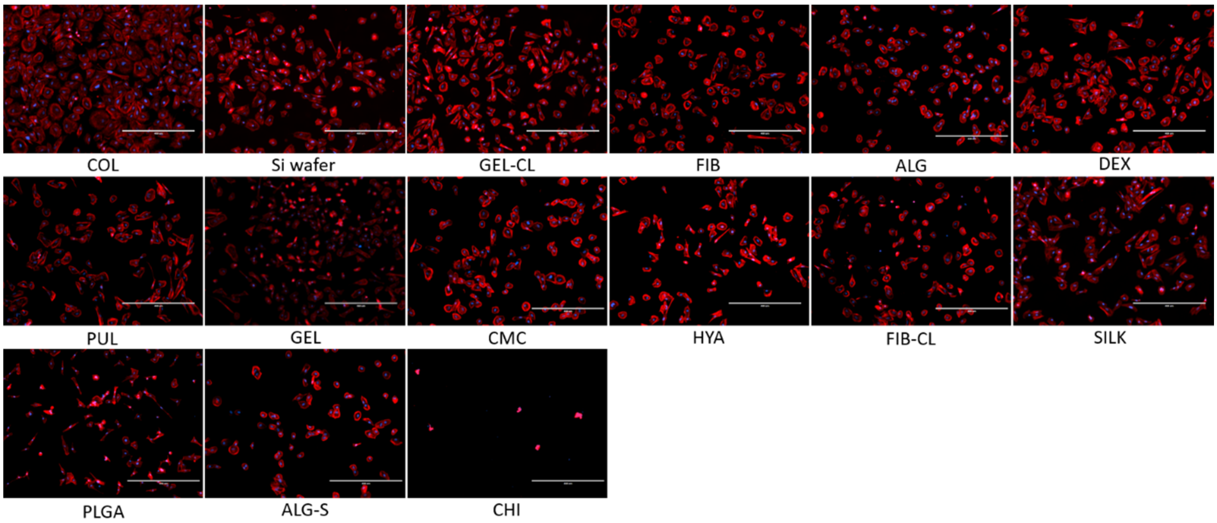

Figure S4. HUIEC cell culture images – ranked.

Figure S4 shows fluorescence micrographs (stained with DAPI and Phalloidin) of HUIEC cells cultured on polymer thin films, ordered according to biocompatibility score.

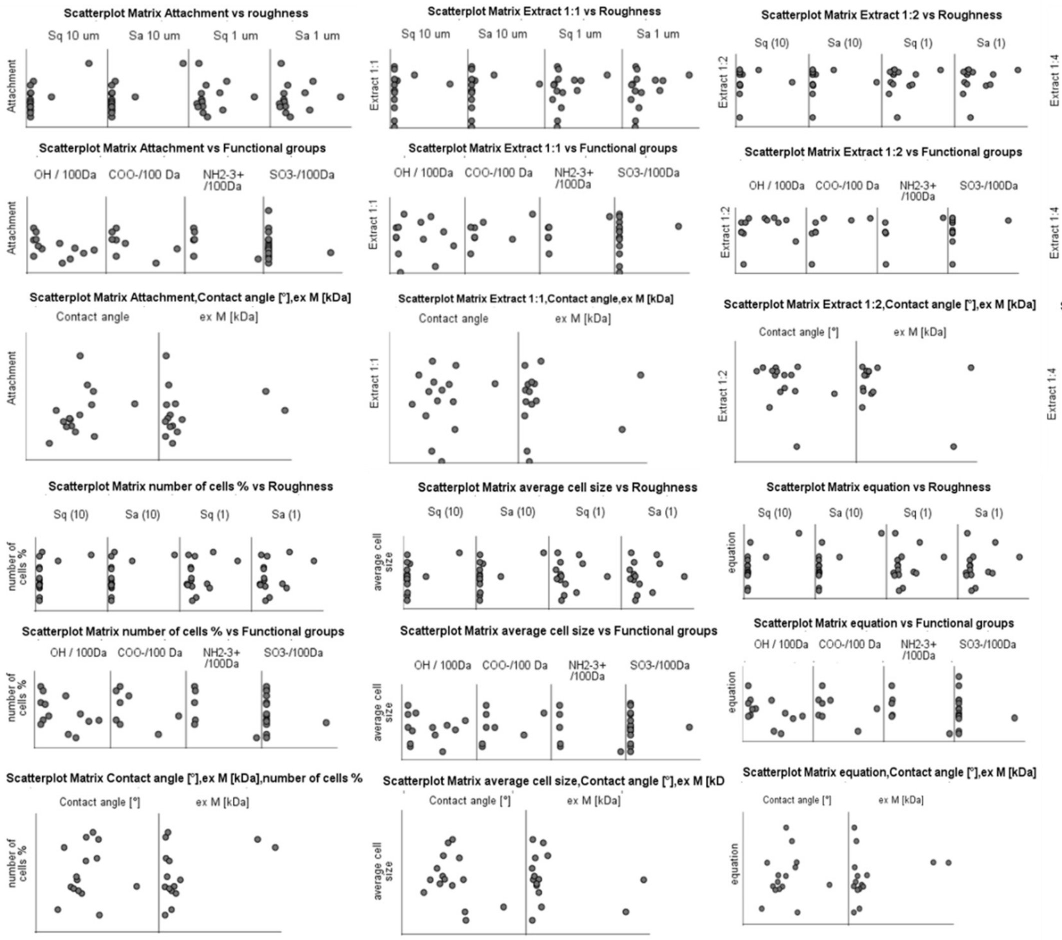

Figure S5. Spearman's nonparametric test results: HUVEC - thin films.

Figure S5 shows scatter plot matrices of HUVEC viability results on varying thin film samples (attachment, extracts), compared to polymer characteristics.

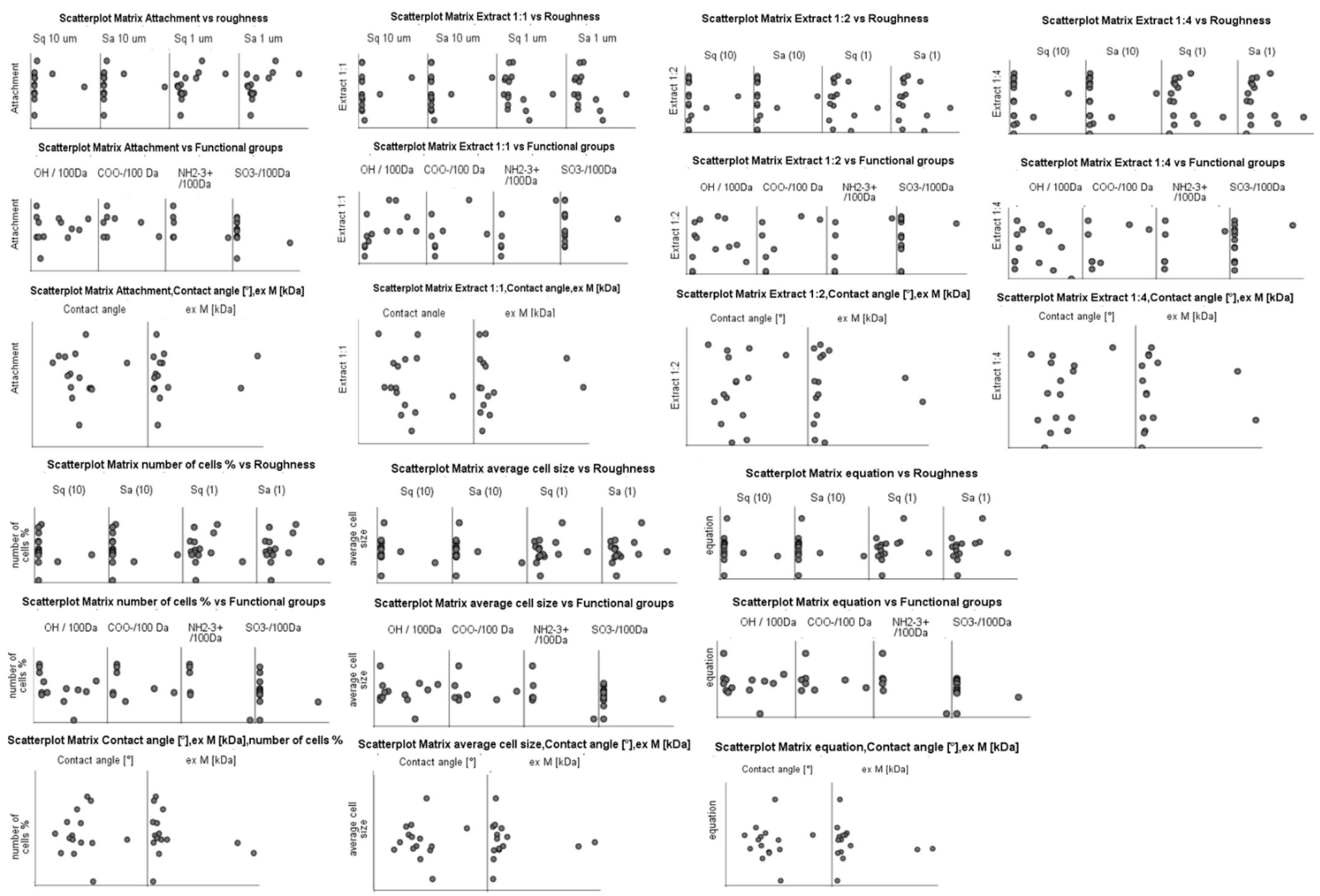

Figure S6. Spearman's nonparametric test results: HUIEC - thin films.

Figure S6 shows scatter plot matrices of HUIEC viability results on varying thin film samples (attachment, extracts), compared to polymer characteristics.

Table S1: Spearman's nonparametric test of HUVEC viability and polymer characteristics

Table S1.1: Spearman's nonparametric test of correlation between HUVEC viability (MTT assay results) on attachment samples and independent variables.

		Sq (10 μm)	Sa (10 μm)	Sq (1 μm)	Sa (1 μm)	Contact angle [°]	OH / 100Da	COO-/100 Da	NH2-3+ /100Da	SO3- /100Da	ex M [kDa]
Correlation Coefficient	Attachment	0.461	0.436	0.050	0.054	0.464	-0.556	-0.675	-0.309	-0.186	0.030
Sig. (2-tailed)		0.084	0.104	0.860	0.850	0.081	0.048	0.066	0.552	0.508	0.914
N		15	15	15	15	15	13	8	6	15	15

Spearman's nonparametric correlation test showed no significant correlation between Attachment and independent variables ($p > 0.05$).

Table S1.2: Spearman's nonparametric test of correlation between HUVEC viability (MTT assay results) on extract 1:1 samples and independent variables.

		Sq (10 µm)	Sa (10 µm)	Sq (1 µm)	Sa (1 µm)	Contact angle [°]	OH / 100Da	COO-/100 Da	NH2-3+ /100Da	SO3- /100Da	ex M [kDa]
Correlation Coefficient	Extract 1:1	0.336	0.364	0.304	0.368	0.129	-0.080	0.405	0.463	0.247	0.211
Sig. (2-tailed)		0.221	0.182	0.271	0.177	0.648	0.794	0.319	0.355	0.374	0.450
N		15	15	15	15	15	13	8	6	15	15

Spearman's nonparametric correlation test showed no significant correlation between Extract 1:1 and independent variables ($p > 0.05$).

Table S1.3: Spearman's nonparametric test of correlation between HUVEC viability (MTT assay results) on extract 1:2 samples and independent variables.

		Sq (10 µm)	Sa (10 µm)	Sq (1 µm)	Sa (1 µm)	Contact angle [°]	OH / 100Da	COO-/100 Da	NH2-3+ /100Da	SO3- /100Da	ex M [kDa]
Correlation Coefficient	Extract 1:2	0.032	0.082	0.146	0.157	-0.332	0.293	.896	0.000	0.124	0.081
Sig. (2-tailed)		0.909	0.771	0.603	0.576	0.226	0.331	0.003	1.000	0.660	0.775
N		15	15	15	15	15	13	8	6	15	15

Spearman's nonparametric correlation test showed significant correlation between Extract 1:2 and functional group COO–/100 Da ($\varrho = 0.896$; $p = 0.003$). There is no significant correlation between other variables.

Table S1.4: Spearman's nonparametric test of correlation between HUVEC viability (MTT assay results) on extract 1:4 samples and independent variables.

		Sq (10 µm)	Sa (10 µm)	Sq (1 µm)	Sa (1 µm)	Contact angle [°]	OH / 100Da	COO-/100 Da	NH2-3+ /100Da	SO3- /100Da	ex M [kDa]
Correlation Coefficient	Extract 1:4	0.207	0.196	0.332	0.350	-0.093	0.006	0.442	0.370	0.371	0.444
Sig. (2-tailed)		0.459	0.483	0.226	0.201	0.742	0.986	0.273	0.470	0.173	0.098
N		15	15	15	15	15	13	8	6	15	15

Spearman's nonparametric correlation test showed no significant correlation between Extract 1:4 and independent variables ($p>0.05$).

Table S1.5: Spearman's nonparametric test of correlation between HUVEC cell number and independent variables.

		Sq (10 µm)	Sa (10 µm)	Sq (1 µm)	Sa (1 µm)	Contact angle [°]	OH / 100Da	COO-/100 Da	NH2-3+ /100Da	SO3- /100Da	ex M [kDa]
Correlation Coefficient	number of cells %	0.407	0.389	0.032	0.032	0.229	-0.465	-0.295	-0.463	-0.247	0.063
Sig. (2-tailed)		0.132	0.152	0.909	0.909	0.413	0.110	0.479	0.355	0.374	0.825
N		15	15	15	15	15	13	8	6	15	15

Spearman's nonparametric correlation test showed no significant correlation between number of cells % and independent variables ($p > 0.05$).

Table S1.6: Spearman's nonparametric test of correlation between HUVEC cell size and independent variables.

		Sq (10 µm)	Sa (10 µm)	Sq (1 µm)	Sa (1 µm)	Contact angle [°]	OH / 100Da	COO-/100 Da	NH2-3+ /100Da	SO3- /100Da	ex M [kDa]
Correlation Coefficient	average cell size	0.150	0.229	-0.182	-0.161	-0.264	-0.185	0.479	-.926	0.062	-0.140
Sig. (2-tailed)		0.594	0.413	0.516	0.567	0.341	0.544	0.230	0.008	0.827	0.620
N		15	15	15	15	15	13	8	6	15	15

Spearman's nonparametric correlation test showed significant correlation between average cell size and functional group NH2-3+/100 Da ($\varrho = -0.926$. $p = 0.008$).

Table S1.7: Spearman's nonparametric test of correlation between weighed HUVEC viability (biocompatibility score) and independent variables.

		Sq (10 μm)	Sa (10 μm)	Sq (1 μm)	Sa (1 μm)	Contact angle [°]	OH / 100Da	COO-/100 Da	NH2-3+ /100Da	SO3- /100Da	ex M [kDa]
Correlation Coefficient	Equation	0.500	0.486	0.071	0.086	0.246	-0.537	-0.295	-0.463	-0.247	0.132
Sig. (2-tailed)		0.058	0.066	0.800	0.761	0.376	0.059	0.479	0.355	0.374	0.638
N		15	15	15	15	15	13	8	6	15	15

Spearman's nonparametric correlation test showed no significant correlation between Equation and independent variables ($p > 0.05$).

Table S2: Spearman's nonparametric test of HUIEC viability and polymer characteristics.

Table S2.1: Spearman's nonparametric test of correlation between HUIEC viability (MTT assay results) on attachment samples and independent variables.

		Sq 10 μm	Sa 10 μm	Sq 1 μm	Sa 1 μm	Contact angle [°]	ex M [kDa]	OH / 100Da	COO^-/100 Da	NH2-3+ /100Da	SO^{3-}/100Da
Correlation Coefficient	Attachment	0.375	0.361	0.464	0.464	-0.354	0.150	-0.100	0.147	-0.617	-0.386
Sig. (2-tailed)		0.168	0.187	0.081	0.081	0.196	0.593	0.746	0.728	0.192	0.193
N		15	15	15	15	15	15	13	8	6	13

Spearman's nonparametric correlation test showed no significant correlation between attachment and independent variables ($p > 0.05$).

Table S2.2: Spearman's nonparametric test of correlation between HUIEC viability (MTT assay results) on extract 1:1 samples and independent variables.

		Sq 10 μm	Sa 10 μm	Sq 1 μm	Sa 1 μm	Contact angle [°]	ex M [kDa]	OH / 100Da	COO^-/100 Da	NH2-3+ /100Da	SO^{3-}/100Da
Correlation Coefficient	Extract 1:1	-0.068	-0.139	-0.146	-0.154	-0.211	-0.039	.681*	0.258	.926*	0.077
Sig. (2-tailed)		0.810	0.621	0.603	0.585	0.451	0.889	0.010	0.538	0.008	0.802
N		15	15	15	15	15	15	13	8	6	13

*Spearman's nonparametric correlation test showed significant correlation between Extract 1:1 and functional group NH2-3+/100 Da ($\varrho = 0.92$, $p = 0.008$). Significant correlation was also found between Extract 1:1 and functional group OH/100 Da ($\varrho = 0.681$, $p = 0.010$).

Table S2.3: Spearman's nonparametric test of correlation between HUIEC viability (MTT assay results) on extract 1:2 samples and independent variables.

		Sq 10 μm	Sa 10 μm	Sq 1 μm	Sa 1 μm	Contact angle [°]	ex M [kDa]	OH / 100Da	COO^-/100 Da	NH2-3+ /100Da	SO^{3-}/100Da
Correlation Coefficient	Extract 1:2	0.121	0.114	0.068	0.071	0.111	0.170	0.296	0.356	.926*	0.154
Sig. (2-tailed)		0.666	0.685	0.810	0.800	0.694	0.545	0.326	0.387	0.008	0.615
N		15	15	15	15	15	15	13	8	6	13

*Spearman's nonparametric correlation test showed significant correlation between Extract 1:2 and functional group NH2-3+/100 Da ($\varrho = 0.926$. $p = 0.008$).

Table S2.4: Spearman's nonparametric test of correlation between HUIEC viability (MTT assay results) on extract 1:4 samples and independent variables.

		Sq 10 μm	Sa 10 μm	Sq 1 μm	Sa 1 μm	Contact angle [°]	ex M [kDa]	OH / 100Da	COO^-/100 Da	NH2-3+ /100Da	SO^{3-}/100Da
Correlation Coefficient	Extract 1:4	0.207	0.211	0.118	0.150	0.275	0.329	-0.111	-0.037	.833*	0.309

Sig. (2-tailed)		0.459	0.451	0.676	0.594	0.321	0.231	0.719	0.931	0.039	0.305
N		15	15	15	15	15	15	13	8	6	13

Spearman's nonparametric correlation test showed significant correlation between Extract 1:4 and functional group NH2-3+/100 Da (ϱ = 0.833. $p = 0.039$).

Table S2.5: Spearman's nonparametric test of correlation between HUIEC cell number and independent variables.

		**Sq 10 µm**	**Sa 10 µm**	**Sq 1 µm**	**Sa 1 µm**	**Contact angle [°]**	**ex M [kDa]**	**OH / 100Da**	**COO^-/100 Da**	**NH2-3+ /100Da**	**SO^{3-}/100Da**
Correlation Coefficient	Number of cells (%)	-0.125	-0.088	-0.097	-0.095	-0.102	-0.252	-0.381	-0.019	-.926	-0.386
Sig. (2-tailed)		0.657	0.756	0.732	0.737	0.718	0.364	0.199	0.965	0.008	0.192
N		15	15	15	15	15	15	13	8	6	13

Spearman's nonparametric correlation test showed significant correlation between Number of cells (%) and functional group NH2-3+/100 Da (ϱ = −0.926. $p = 0.008$).

Table S2.6: Spearman's nonparametric test of correlation between HUIEC cell size and independent variables.

		**Sq 10 µm**	**Sa 10 µm**	**Sq 1 µm**	**Sa 1 µm**	**Contact angle [°]**	**ex M [kDa]**	**OH / 100Da**	**COO^-/100 Da**	**NH2-3+ /100Da**	**SO^{3-}/100Da**
Correlation Coefficient	average cell size	-0.161	-0.121	0.050	0.014	-0.221	0.066	0.177	-0.074	-0.463	-0.154
Sig. (2-tailed)		0.567	0.666	0.860	0.960	0.428	0.815	0.563	0.862	0.355	0.615
N		15	15	15	15	15	15	13	8	6	13

Spearman's nonparametric correlation test showed no significant correlation between average cell size and independent variables ($p > 0.05$).

Table S2.7: Spearman's nonparametric test of correlation between weighed HUIEC viability (biocompatibility score) and independent variables.

		**Sq 10 µm**	**Sa 10 µm**	**Sq 1 µm**	**Sa 1 µm**	**Contact angle [°]**	**ex M [kDa]**	**OH / 100Da**	**COO^-/100 Da**	**NH2-3+ /100Da**	**SO^{3-} /100Da**
Correlation Coefficient	Equation	0.000	0.007	0.193	0.196	-0.225	-0.011	-0.113	-0.110	-0.772	-0.386
Sig. (2-tailed)		1.000	0.980	0.491	0.483	0.420	0.970	0.712	0.795	0.072	0.193
N		15	15	15	15	15	15	13	8	6	13

Spearman's nonparametric correlation test showed no significant correlation between Equation and independent variables ($p > 0.05$).
